# Manual Acupuncture at PC6 Ameliorates Acute Restraint Stress-Induced Anxiety in Rats by Normalizing Amygdaloid Noradrenergic Response

**DOI:** 10.1155/2017/4351723

**Published:** 2017-08-16

**Authors:** ZhengLin Zhao, Sang Chan Kim, HongFeng Liu, Jie Zhang, YuHua Wang, Il Je Cho, Bong Hyo Lee, Chang Hyun Song, Chul Won Lee, Chae Ha Yang, RongJie Zhao, YiYan Wu

**Affiliations:** ^1^School of Mental Health, Qiqihar Medical University, Qiqihar 161006, China; ^2^Department of Pharmacology, Mudanjiang Medical University, Mudanjiang 157011, China; ^3^College of Oriental Medicine, Daegu Haany University, Daegu 706-060, Republic of Korea

## Abstract

Acupuncture improves ethanol withdrawal-induced anxiety in rats in an acupoint-dependent manner. Thus, the present study investigated the effects of acupuncture on acute restraint stress- (ARS-) induced anxiety. Male rats were exposed to ARS for 3 h followed by acupuncture at either PC6 (Neiguan), HT7 (Shenmen), or a nonacupoint (tail) once a day for three consecutive days. Five minutes after the third acupuncture treatment, anxiety-like behavior was evaluated in an elevated plus maze (EPM). Additionally, plasma corticosterone (CORT) levels were measured by radioimmunoassay and the concentrations of norepinephrine (NE) and 3-methoxy-4-hydroxy-phenylglycol (MHPG) in the central nucleus of the amygdala (CeA) were determined using high-performance liquid chromatography. Acupuncture at PC6, but not HT7 or a nonacupoint, attenuated anxiety-like behavior, but this attenuation was abolished by a postacupunctural intra-CeA infusion of NE. Acupuncture at PC6 also reduced the oversecretion of plasma CORT and inhibited increases in amygdaloid NE and MHPG induced by ARS. Further, Western blot analyses and real-time polymerase chain reaction assays revealed that acupuncture at PC6 prevented ARS-induced enhancements in the protein and mRNA expressions of tyrosine hydroxylase in the CeA. These results suggest that acupuncture performed specifically at acupoint PC6 reduces ARS-induced anxiety-like behavior by dampening amygdaloid noradrenergic responses.

## 1. Introduction

Stress-related mental disorders such as depression and anxiety are highly prevalent in today's society. In experimental studies, acute restraint stress (ARS) is an emotional stressor that activates various neurochemical, physiological, endocrinological, cognitive, and behavioral responses. In rats, ARS increases plasma corticosterone (CORT) levels [1] and causes an imbalance in the hippocampal redox state [2]. ARS also disrupts recognition memory retrieval in rodent object recognition and object location tasks [3] and interferes with effort-related decision-making associated with corticotrophin-releasing factor (CRF) in the brain stem [4]. If the intensity and duration of these biochemical and functional disturbances are sufficiently severe, then the brain cannot return to normal functioning and behaviors similar to those of depression and anxiety may arise.

The central nucleus of the amygdala (CeA) is a major structure involved in the processing and expression of emotional information. The CeA is heavily innervated by CRF neurons that widely project to mood-related limbic regions and play key roles in the mediation of fear and anxiety [5]. ARS increases the expression of CRF mRNA in the CeA [6], and intracerebroventricular administration of CRF elicits anxiety-like behavior in rats that is blocked by intra-CeA infusion of CRF antagonists [7, 8]. The CeA also receives dense projections of noradrenergic fibers from the locus coeruleus (LC) and the nucleus tractus solitarii (NTS) and, accordingly, amygdaloid norepinephrine (NE) also modulates stress-related anxiety responses. In vivo microdialysis and pharmacological studies have shown that ARS increases amygdaloid NE release [9], while intra-CeA administration of alpha-1 noradrenergic receptor antagonists prevents ARS-induced anxiety-like behavior in rats [10]. Moreover, NE is a CRF secretagogue; increased amygdaloid NE levels during ethanol withdrawal parallel the increased expression of CRF mRNA in the CeA [11].

Acupuncture has long been used in traditional Chinese medicine (TCM) for the treatment of various stress-related mental disorders, including depression and anxiety. Acupuncture can ameliorate stress-induced biochemical and physiological imbalances in the central nervous system and improve related symptoms. For example, acupuncture at acupoint ST36 (Zusanli) attenuates chronic stress-induced depression-like symptoms by modulating the hypothalamic-pituitary-adrenal (HPA) axis [12] and acupuncture at acupoint GV20 (Baihui) improves cognitive function in a rodent model of cerebral ischemia by reducing the production of hippocampal reactive oxygen species [13]. Furthermore, Kim et al. demonstrated that acupuncture at acupoint PC6 (Neiguan) alleviates chronic mild stress-induced anxiety-like behavior in rats by suppressing hypothalamic c-Fos expression [14], and our research group found that acupuncture at acupoint HT7 (Shenmen) ameliorates ethanol withdrawal-induced anxiety by normalizing amygdaloid catecholamine levels [11].

Thus, to further current knowledge regarding the therapeutic efficacy of acupuncture for stress-induced mental disorders, the present study evaluated the effects of acupuncture on ARS-induced anxiety and investigated the possible involvement of the CeA NE system in this process.

## 2. Materials and Methods 

### 2.1. Animals and Experimental Design

Adult male Sprague-Dawley rats (250–270 g) were obtained from the Laboratory Animal Center at Mudanjiang Medical University (Mudanjiang, China). The rats were individually housed, provided food and water ad libitum, and maintained on a 12 h light/dark cycle throughout the course of the study. All animal procedures were performed in accordance with the National Institutes of Health guidelines concerning the Care and Use of Laboratory Animals and approved by the Animal Care and Use Committee of Mudanjiang Medical University.

**Figure 1:**
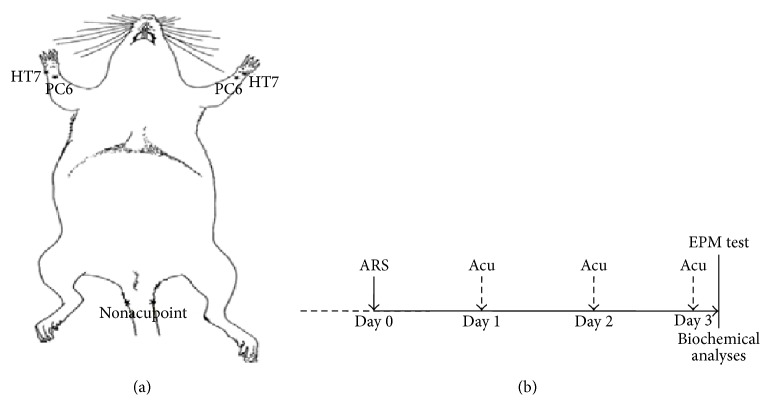
(a) Diagram of the acupoint PC6, HT7 and the nonacupoint located in the tail in a rat. (b) Time schedule for the study of therapeutic effects of acupuncture on ARS-induced anxiety.

Prior to the experiment, all rats were habituated to human handling by being gently picked up and held for 2 min each day for 7 days in their home room. Next, they were randomly assigned to either ARS or non-ARS groups. On the day of the experiment, each rat in the ARS groups was transported to an experimental room and individually placed into a Plexiglas tube (length: 19 cm, diameter: 5 cm) for 3 h at room temperature; all stress experiments began at 8:30 A.M. Immediately after the ARS procedure, the rats were returned to their home cages, where they stayed for 72 h. During this 3-day period, each rat was bilaterally treated with acupuncture for 1 min once daily at either acupoints HT7 or PC6 or a nonacupoint on the tail; thus, there were a total of three treatments (Figures 1(a) and 1(b)). The acupoints used to treat the rats in the present study were equivalent to those in human male subjects and in animal acupuncture references.

The acupuncture stimulation involved the insertion of stainless steel needles (diameter: 0.2 mm) into either the HT7 or PC6 acupoint or the tail nonacupoint at a depth of 2-3 mm; the needles were manipulated using the reduction and reinforcement method (for additional details see [11]). The sham acupuncture treatment, performed on two groups of rats that either did or did not undergo ARS, involved holding each rat for 1 min without the insertion of acupuncture needles. This sham procedure replicated the immobilization experienced by the acupuncture-treated rats and did not generate any significant behavioral or neurochemical effects (data not shown).

Five minutes after the third acupuncture (or sham) treatment, all rats were individually tested in an elevated plus maze (EPM, Yishu Co., Shanghai, China) to measure their responses to an anxiogenic setting. Briefly, without any pretest handling, each rat was placed in the center of the maze and the cumulative time spent in each arm and the number of entries into the open or closed arms were recorded during a 5 min test session. Entry into an arm was defined as beginning when the animal had placed all four paws in that particular arm; time spent inside the center portion (10 cm × 10 cm) of the maze was not considered. The maze was cleaned with water after each rat had been tested and exploration of the open arms was encouraged by dim light (2 × 60 W). Behavior in the maze was recorded by a video tracking system. Data were recorded as time spent in open arms and expressed as a percentage of total time spent in the arms, which was calculated as follows:

(1) Percentage  of  Tspent  in  open  arms=Tspent  in  open  armsTspent  in  closed  arms+Tspent  in  open  arms.

Immediately following the EPM test, the rats were euthanized and decapitated. Blood samples were collected in a chilled tube containing EDTA (20 mg/mL, 20 *μ*L) and centrifuged at 1,000 ×g and 4°C for 10 min to separate plasma for the plasma CORT assay. Next, the entire brain was removed and stored at −80°C until tissue samples from the CeA were punched out based on the CeA coordinates: anterior-posterior (AP) = −2.2 mm, medial-lateral (ML) = 4.3 mm, and dorsal-ventral (DV) = −8.0 mm (Paxinos and Watson [15]). The samples were analyzed using high-performance liquid chromatography (HPLC), a real-time polymerase chain reaction (PCR) assay, and Western blot analyses.

### 2.2. Radioimmunoassay (RIA) of Plasma CORT

CORT levels in the plasma samples were measured with the ImmuChem™ double antibody ^125^I CORT RIA kit (MP Biomedicals; Orangeburg, NY, USA) according to the manufacturer's instructions, and the results are expressed as ng/mL of plasma. Briefly, the rat plasma was diluted (1 : 200) with the steroid diluent, incubated with ^125^I CORT and anti-CORT (antibody) for 2 h at room temperature, and then centrifuged at 1,500 ×g and 4°C for 20 min to separate the antibody-bound ^125^I CORT. Finally, the supernatant was carefully aspirated, and the radioactivity of remaining pellets was measured in a gamma counter [16].

### 2.3. High-Performance Liquid Chromatography (HPLC) Analyses of NE and Its Metabolites in the CeA

Frozen CeA tissues were weighted, sonicated in 0.1 mol/L HClO_4_, and centrifuged at 26,000 ×g and 4°C for 15 min, and 20 *μ*L of supernatant was injected directly into HPLC medium and analyzed using an electrochemical detector (Coulochem II, ESA; Bedford, MA, USA). The HPLC system consisted of a C-18 reverse-phase column (5 *μ*m ODS, Altex; Ann Arbor, MI, USA) and an electrochemical transducer with a glassy carbon electrode set at 350 mV. The mobile phase consisted of 0.163 mol/L citric acid, 0.02 mmol/L EDTA, 0.69 mmol/L sodium octanesulfonic acid as an ion-pairing reagent, 4% (v/v) acetonitrile, and 1.7% (v/v) tetrahydrofuran and was titrated to pH 3.0 with H_3_PO_4_. The peaks and values of NE and 3-methoxy-4-hydroxy-phenylglycol (MHPG) in the samples were identified and calculated by comparing their retention times and peak heights with those of standards; the results are expressed as ng/g wet tissue.

### 2.4. Western Blot Analyses

Frozen CeA tissue samples were homogenized in lysis buffer [20 mM Tris, 5 mM MEDTA, 1% Nonidet P-40 (vol/vol), and protease inhibitors], incubated on ice for 20 min, and centrifuged at 19,000 ×g and 4°C for 20 min. Next, the supernatants were resolved by electrophoresis on a 12% sodium dodecyl sulfate-polyacrylamide gel and the proteins were transferred onto a nitrocellulose membrane (Schleicher & Schuell GmbH, Dassel, Germany). The membrane was then incubated with either an anti-mouse tyrosine hydroxylase (TH) antibody or an anti-goat *β*-actin antibody (Santa Cruz Biotechnology; Santa Cruz, CA, USA), washed in Tris-buffered saline with Tween-20 (TBST; 10 mM Tris-Cl, pH 7.5, 150 mM NaCl, and 0.05% Tween-20), and incubated for 1 h with the appropriate peroxidase-conjugated secondary antibodies. Bands corresponding to TH and *β*-actin were visualized using enhanced chemiluminescence Western blot detection reagents (Amersham Biosciences, Piscataway, NJ, USA).

### 2.5. Real-Time PCR Analysis

Total RNA was extracted from the CeA tissue samples using a Trizol reagent (Invitrogen, Carlsbad, CA) and the cDNA was synthesized by reverse transcription using an oligo (dT) primer. Then, a real-time PCR assay was performed using a LightCycler 1.5 (Roche, Mannheim, Germany) and the LightCycler DNA Master SYBR green-I kit according to the manufacturer's instructions. The primers (Bioneer Corporation, Daejeon, Republic of Korea) were 5′-ATGCCCACCCCCAGCGCCCC-3′ (sense) and 5′-GACACTTTTCTTGGGAACCA-3′ (antisense) for TH and 5′-GTCGTACCACTGGCATTGTG-3′ (sense) and 5′-GCCATCTCTTGCTCGAAGTC-3′ (antisense) for *β*-actin. The housekeeping gene *β*-actin was used as an endogenous reference and the relative expression levels of TH mRNA were calculated using the following formula: ΔCT = CT (TH) −  CT (*β*-actin); ΔΔCT = ΔCT (treated) −  ΔCT (saline). The levels were expressed as 2^−ΔΔCT^.

### 2.6. Microinjections of NE into the CeA

For the microinjection procedure, rats were anesthetized by intraperitoneal injection of sodium pentobarbital (50 mg/kg). Stainless steel guide cannulae (15 mm; 23 gauge) were bilaterally implanted into the brain using a stereotaxic instrument so that the cannula tips were 2 mm above the CeA (the coordinates as described above). To determine whether the anxiolytic effects of acupuncture treatment were mediated by the amygdaloid NE system, NE was dissolved in modified Ringer's solution (150 mM NaCl, 3.0 mM KCl, 1.4 mM CaCl2, and 0.8 mM MgCl2 in 10 mM phosphate buffer at pH 7.4) and bilaterally delivered through the injectors (internal cannulae, 28 gauge, 2 mm longer than the guide cannulae, therefore, exactly targets the CeA) over 1 min into the CeA at a concentration of 10 nmol/100 nL/side using motorized syringe pumps (Sage Instruments; Boston, MA, USA) 5 min after the third acupuncture treatment. Two minutes after the NE infusion, the rats were tested in the EPM (Yishu Co.) using the procedure described above. Immediately after the EPM test, the rats were decapitated and their brains were removed to verify the placements of the guide cannulae.

### 2.7. Statistical Analysis

All data are expressed as means ± standard errors of the mean (SEM) and were analyzed with a one-way analysis of variance (ANOVA) followed by the Newman–Keuls (NK) multiple comparisons test. Commercially available GraphPad Prism 5.0 software (GraphPad Software, San Diego, CA, USA) was used to perform the statistical analyses and *p* values < 0.05 were considered to indicate statistical significance. The normality of data was checked and the homogeneity of variances was analyzed by Bartlett's test before each running of a one-way ANOVA.

## 3. Results

### 3.1. Effects of Acupuncture on Anxiety-Like Behavior and the Involvement of Amygdaloid NE

**Figure 2:**
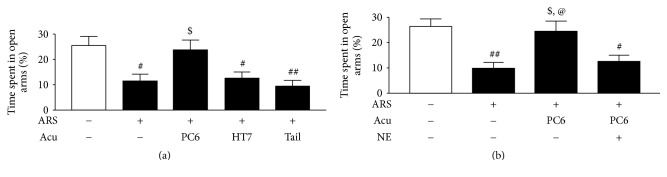
Effects of acupuncture on anxiety-like behavior and the involvement of amygdaloid NE. Data are expressed as mean ± SEM (*n* = 8) of the percentages of time spent in the open arms of the EPM for a 5 min test period. (a) Anxiolytic effects of acupuncture at PC6. (b) The involvement of amygdaloid NE in anxiolytic effects of acupuncture. ^#^*p* < 0.05, ^##^*p* < 0.01, compared with non-ARS control group; ^$^*p* < 0.05, compared with ARS control group; ^@^*p* < 0.05, compared with ARS + PC6 + NE group.

The presence of anxiety-like behavior was evident in rats 3 days after they experienced ARS, as shown by shorter time spent in the open arms during the EPM test [ARS control (*n* = 8) versus non-ARS control group (*n* = 8), *F*_(4,35)_ = 5.71, *p* < 0.01, NK: *p* < 0.05]. However, rats that received acupuncture treatment at PC6 (*n* = 8) (*p* < 0.05) spent a significantly greater percentage of time in the open arms compared to the ARS control group. The percentage of time spent in the open arms by the HT7 group (*n* = 8) (*p* > 0.05) and the tail (nonacupoint) group (*n* = 8) (*p* > 0.05) did not significantly differ from that of the ARS control group, indicating anxiolytic effects of acupuncture at PC6 and the importance of acupuncture treatment location in producing anxiolytic effects (Figure 2(a)).

Postacupunctural intra-CeA administration of NE (*n* = 8) almost completely abolished the anxiolytic effects of acupuncture at PC6 as evidenced by behavior in the EPM, *F*_(3,20)_ = 7.23, *p* < 0.01 (Figure 2(b)). These results imply that the anxiolytic effects of acupuncture treatment at PC6 were mediated by amygdaloid NE.

### 3.2. Effects of Acupuncture at PC6 on Plasma CORT Levels after ARS

**Figure 3:**
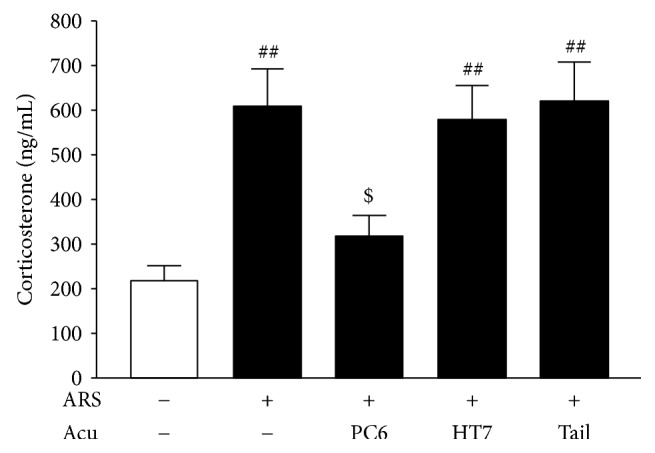
Effects of acupuncture at PC6 on plasma corticosterone (CORT) levels. Data are expressed as mean ± SEM (*n* = 8) of the concentrations of plasma CORT. ^##^*p* < 0.01, compared with non-ARS control group; ^$^*p* < 0.05, compared with ARS control group.

Plasma CORT was measured by RIA to neurochemically confirm the anxiolytic effects of acupuncture at PC6. Three days after ARS, the plasma CORT levels of the ARS control group (*n* = 8) were significantly higher than those of the non-ARS control group (*n* = 8), *F*_(4,35)_ = 7.61, *p* < 0.001 (NK: *p* < 0.01). In agreement with the behavioral results, acupuncture treatment at PC6 (*n* = 8) significantly attenuated the ARS-induced increases in plasma CORT levels compared to the ARS control group (*p* < 0.05). On the other hand, the plasma CORT levels of the HT7 (*n* = 8) (*p* > 0.05) and tail (nonacupoint) groups (*n* = 8) (*p* > 0.05) did not significantly differ from those in the ARS control group (Figure 3).

### 3.3. Effects of Acupuncture on NE and MHPG Levels in the CeA

**Figure 4:**
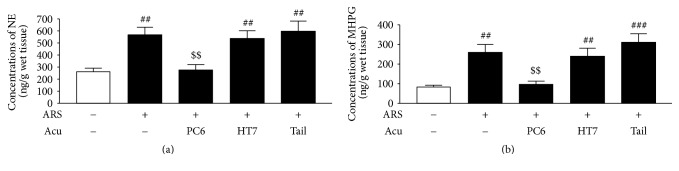
Effects of acupuncture on (a) NE and (b) MHPG levels in the CeA. Data are expressed as mean ± SEM (*n* = 7) of the concentrations of NE or MHPG. ^##^*p* < 0.01; ^###^*p* < 0.001, compared with non-ARS control group; ^$$^*p* < 0.01, compared with ARS control group.

HPLC analysis revealed that the ARS control group (*n* = 7) had significantly higher NE and MHPG levels in the CeA than the non-ARS control group (*n* = 7) 3 days after the stress procedure [NE: *F*_(4,30)_ = 8.03, *p* < 0.001, (NK: *p* < 0.01); MHPG: *F*_(4,30)_ = 10.63, *p* < 0.001, (NK: *p* < 0.01)], but no significant changes in the levels of DA or 3,4-dihydroxyphenylacetic acid (DOPAC) were observed (data not shown). However, there was a significant decrease in the pathophysiologically excessive amygdaloid NE levels in the PC6 group (*n* = 7) (NE: *p* < 0.01, MHPG: *p* < 0.01) compared to the ARS control group. On the other hand, the amygdaloid NE and MHPG levels of the HT7 (*n* = 7) (NE: *p* > 0.05; MHPG: *p* > 0.05) and the tail (nonacupoint) groups (*n* = 7) (NE: *p* > 0.05; MHPG: *p* > 0.05) did not significantly differ from that of the ARS control group (Figure 4).

### 3.4. Effects of Acupuncture at PC6 on TH Protein and mRNA Expressions in the CeA

**Figure 5:**
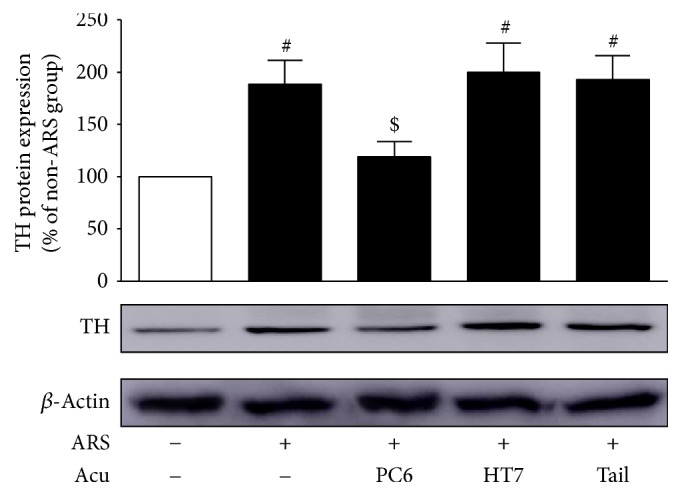
Effects of acupuncture at PC6 on TH protein expressions in the CeA. Data are expressed as mean ± SEM (*n* = 6) of the percentages of non-ARS control group. ^#^*p* < 0.05, compared with non-ARS control group; ^$^*p* < 0.05, compared with ARS control group.

**Figure 6:**
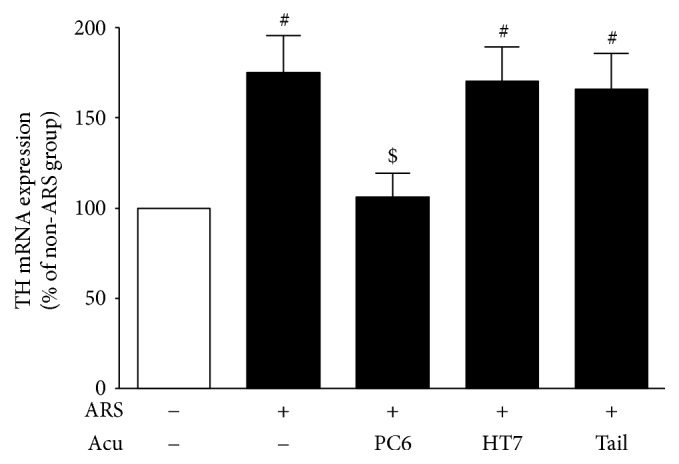
Effects of acupuncture at PC6 on TH mRNA expressions in the CeA. Data are expressed as mean ± SEM (*n* = 6) of the percentages of non-ARS control group. ^#^*p* < 0.05, compared with non-ARS control group; ^$^*p* < 0.05, compared with ARS control group.

Western blot analyses revealed that the ARS control group (*n* = 6) expressed significantly more TH protein in the CeA than the non-ARS control group (*n* = 6) 3 days after stress, *F*_(4,20)_ = 5.36, *p* < 0.01 (NK: *p* < 0.05). Real-time PCR confirmed this result, *F*_(4,20)_ = 5.12, *p* < 0.01, (NK: *p* < 0.05). However, the PC6 group (*n* = 6) exhibited significantly smaller increases in TH protein (*p* < 0.05) and mRNA (*p* < 0.05) expressions compared to the ARS control group. On the other hand, the TH protein and mRNA expressions of the HT7 group (*n* = 6) (both *p* values > 0.05) and tail (nonacupoint) group (*n* = 6) (both *p* values > 0.05) did not significantly differ from those of the ARS control group (Figures 5 and 6).

## 4. Discussion

The present findings demonstrate that acupuncture at PC6, but not HT7 or a nonacupoint in the tail, attenuates ARS-induced anxiety-like behavior in rats. Additionally, acupuncture at PC6 inhibited the oversecretion of plasma CORT and reduced the increased levels of amygdaloid NE and MHPG following ARS. To the best of our knowledge, this is the first experimental evidence showing that acupuncture at the specific acupoint PC6 exerts anxiolytic effects after ARS in rats by inhibiting the amygdaloid noradrenergic system.

ARS can induce anxiety-like behavior that persists for several days after the stressor. Malisch et al. found that ARS significantly changes the wheel-running behavior up to 20 h after the stress in mice [17]. Vila-Verde et al. reported the development of robust anxiety-like behavior in individually housed rats seven days after ARS [18]. Likewise, a preliminary study by our research group found that substantial anxiety-like behavior in individually housed rats persisted for five days after a 3 h exposure to ARS and that this behavior peaked three days after ARS; however, the former finding was not significant. Thus, the present study assessed anxiety-like behavior three days after ARS. In the present study, acupuncture treatment at PC6, but not HT7, significantly increased the percentage of time spent by rats exposed to ARS in the open arms of the EPM three days after the stressor. This finding is consistent with the studies done by our research group and others that acupunctural treatment during ethanol and nicotine withdrawal attenuated withdrawal-induced anxiety [11, 19] and suggests that acupuncture at the specific acupoint PC6 may have anxiolytic effects that could mitigate the development of ARS-induced mental disorders.

The response to ARS encompasses a series of neurochemical, physiological, and morphological changes characteristic of short-term and long-term dysregulation of central neuroendocrine systems that are responsible for ARS-induced mental disorders. For example, ARS-induced increases in glutamate release in rats return to prestress baseline levels in the prefrontal cortex but not the hippocampus at 1 h after the stress [20]; the dendritic remodeling of basolateral amygdaloid neurons in mice begins immediately after ARS and persists for at least 3 days after the stress, and the mice exhibit resistance to fear extinction [21–23]. The HPA axis is highly vulnerable to ARS, and the abnormality of plasma CORT levels is a hallmark of disturbance of the HPA response to stress. It is well documented that ARS increases plasma CORT levels underlying anxiety-like behavior in rodents [24]. In the present study, RIA analyses revealed there were significant increases in plasma CORT levels in the rats three days after ARS when examined immediately following the EPM tests. These results indicate the existence of a disordered HPA functioning even after several recovery days from ARS and also reflect the occurrence of stress sensitization that is argued by Nasca et al. [25] since the EPM test can be considered as a stressful event to rats. In agreement with the results from the behavioral tests, in the present study, the RIA analyses also showed that acupuncture treatment at PC6, but not HT7, in rats that underwent ARS attenuated the ARS-induced oversecretion of plasma CORT. These results not only provide neurochemical support for the inhibitory effects of acupuncture on ARS-induced anxiety but also indicate that the anxiolytic effects are mediated by the ability of acupuncture to improve the derailed HPA functioning induced by ARS.

Converging evidence indicates that overactivation within the amygdaloid noradrenergic system elicits anxiety-like behavior in rats. Systemic administration of yohimbine, an anxiogenic alpha2 receptor antagonist, increases NE release in the CeA [26], while intra-CeA administration of propranolol, a nonselective beta receptor antagonist, blocks isoproterenol-induced enhancements in auditory fear conditioning memory in rats [27]. Thus, the present study assessed the possible involvement of the CeA NE system in the anxiolytic effects of acupuncture at PC6 using neurochemical and behavioral analyses. HPLC revealed marked increases in amygdaloid NE and MHPG levels three days after ARS but these increases were inhibited by acupuncture at PC6. Additionally, the MHPG/NE ratio was greater in ARS rats compared to non-ARS rats (0.46 versus 0.32, resp.), but this was also restored in rats that received acupuncture at PC6 (0.35). In the EPM test, postacupunctural intra-CeA infusions of NE almost completely abolished the anxiolytic effects of acupuncture at PC6. Taken together, these findings suggest that ARS enhances both NE synthesis and utilization in the CeA, that acupuncture normalizes both of them, and that the anxiolytic effects of acupuncture are mediated by specific treatment at acupoint PC6.

TH is the rate-limiting enzyme of catecholamine synthesis. Expressions of TH protein and mRNA in mood-related brain regions are affected by a variety of stressors. For example, both acute and repeated stress increase the mRNA levels of TH in rat LC and NTS [28, 29], and alcohol-abstinent rats exhibit elevated levels of phosphorylated TH in the hypothalamus [30]. In the present study, Western blots revealed a significant increase in TH protein expression in the CeA three days after ARS, indicating increased catecholamine synthesis in the CeA. Because the DA and DOPAC contents in the CeA remained unchanged three days after ARS (data not shown), the enhancement in TH protein expression was likely responsible for the increased synthesis of NE following ARS. To further characterize the relationship between the protein levels and gene transcription of TH, real-time PCR assays were conducted and, in agreement with the TH protein expression analyses, revealed an elevated expression of amygdaloid TH mRNA following ARS. These same Western blot and real-time PCR assays also showed that acupuncture at PC6 significantly attenuates the increased TH protein and mRNA expressions in the CeA. Taken together, these biochemical findings indicate that acupuncture at PC6 produces anxiolytic effects by suppressing the upregulation of amygdaloid NE synthesis induced by ARS.

PC6 and HT7 are two important acupoints in TCM that have frequently been used to treat several types of anxiety [31]. The present study showed that acupuncture at PC6, but not HT7, results in anxiolytic effects by improving the ARS-induced dysregulation of the amygdaloid NE system. Conversely, previous studies from our research group have indicated that acupuncture at HT7, but not PC6, attenuates alcohol withdrawal-induced anxiety by normalizing amygdaloid catecholamine levels [11]. Similar results were reported by Shim et al., who found that acupuncture at HT7, but not PC6, normalizes cocaine-induced TH expression in the VTA [32], whereas acupuncture at PC6 alleviates chronic mild stress-induced anxiety by restoring hypothalamic neuronal activity [14]. According to TCM, PC6 is the Vessel point of the Pericardium Meridian and HT7 is the Source point of the Heart Meridian and the function of the Heart (in TCM) corresponds with the function of the brain (in Western medicine). Although the functional connections of these respective Meridians are empirically delineated in TCM, exact anatomical and physiological profiles of the Meridians have yet to be characterized. Therefore, the discrepancies between PC6 and HT7 regarding treatment for anxiety remain unclear, but these acupoints appear to produce etiology- and mechanism-specific anxiolytic effects.

In summary, acupuncture at the acupoint PC6 attenuated ARS-induced anxiety-like behavior and this attenuation was blocked by a postacupunctural intra-CeA infusion of NE, which inhibited enhanced plasma CORT secretion, and restored the increase in amygdaloid NE and MHPG levels. Additionally, acupuncture at PC6 normalized ARS-induced increases in both the TH protein and mRNA expressions in the CeA. Taken together, these findings suggest that acupuncture at PC6 exerts therapeutic effects on ARS-induced anxiety by dampening overproduction of amygdaloid NE.
